# Obesity Enhances Disease Severity in Female Mice Following West Nile Virus Infection

**DOI:** 10.3389/fimmu.2021.739025

**Published:** 2021-08-31

**Authors:** Elizabeth Geerling, E. Taylor Stone, Tara L. Steffen, Mariah Hassert, James D. Brien, Amelia K. Pinto

**Affiliations:** Department of Molecular Microbiology and Immunology, Saint Louis University, St. Louis, MO, United States

**Keywords:** obesity, chronic inflammation, viral infection, West Nile virus, neutralizing antibody, vaccination, sex difference

## Abstract

A rise in adiposity in the United States has resulted in more than 70% of adults being overweight or obese, and global obesity rates have tripled since 1975. Following the 2009 H1N1 pandemic, obesity was characterized as a risk factor that could predict severe infection outcomes to viral infection. Amidst the SARS-CoV-2 pandemic, obesity has remained a significant risk factor for severe viral disease as obese patients have a higher likelihood for developing severe symptoms and requiring hospitalization. However, the mechanism by which obesity enhances viral disease is unknown. In this study, we utilized a diet-induced obesity mouse model of West Nile virus (WNV) infection, a flavivirus that cycles between birds and mosquitoes and incidentally infects both humans and mice. Likelihood for severe WNV disease is associated with risk factors such as diabetes that are comorbidities also linked to obesity. Utilizing this model, we showed that obesity-associated chronic inflammation increased viral disease severity as obese female mice displayed higher mortality rates and elevated viral titers in the central nervous system. In addition, our studies highlighted that obesity also dysregulates host acute adaptive immune responses, as obese female mice displayed significant dysfunction in neutralizing antibody function. These studies highlight that obesity-induced immunological dysfunction begins at early time points post infection and is sustained through memory phase, thus illuminating a potential for obesity to alter the differentiation landscape of adaptive immune cells.

## Introduction

Following the 2009 H1N1 pandemic, a link between obesity and enhanced viral infection severity first came to light. Similarly, amidst the SARS-CoV-2 pandemic, obesity has been cited as a risk factor for SARS-CoV-2 patients to develop coronavirus disease 2019 (COVID-19) (1, 2). Obese COVID-19 patients also have a higher likelihood for requiring hospitalization due to severe symptoms, in addition to a higher mortality rate when compared to healthy weight patients (3, 4). While multiple studies have begun to address the link between obesity and severe disease (5–8), the mechanism by which obesity heightens the likelihood of severe disease outcome is still unclear.

Within the United States, ~72% of adults are overweight, while ~40% are characterized as obese based upon a body mass index greater than or equal to 30 kg/m^2^ (9). Half of United States adults are predicted to be obese by 2030 (10). Globally, obesity rates have tripled since 1975 resulting in 1 out of 3 people being currently classified as overweight or obese (11, 12). Rising obesity rates are problematic due to obesity being linked to numerous comorbidities including nonalcoholic fatty liver disease, type 2 diabetes and respiratory distress, as well as being a risk factor for metabolic syndrome (13–15). Further, within the obese state, energy intake often exceeds energy expenditure, resulting in a positive energy balance that can result in fat accumulation. This accumulation can cause adipocyte enlargement, thus interfering with blood supply to adipocytes and inducing a hypoxic state. Hypoxia within adipose tissue can incite necrosis and result in macrophage infiltration, leading to the production of pro-inflammatory cytokines like interleukin-1β (IL-1β) and tumor necrosis factor-α (TNF-α) that contribute to a state of chronic inflammation seen in obese subjects (16, 17).

Retrospective analyses following the 2009 H1N1 pandemic classified obesity as an independent risk factor of severe H1N1 infection outcomes, as a large proportion of hospitalized patients who succumbed to H1N1 infection were obese (18–23). Since the 2009 H1N1 pandemic highlighted the susceptibility of the obese population to severe infection outcomes, numerous laboratories sought to determine if increasing vaccination rates of this high-risk population could mitigate the risk for severe disease. Such studies, predominately done utilizing respiratory viruses, have revealed that obese humans and mice displayed impaired immune responses to vaccination (8, 24–27) and classified obesity as a comorbidity that exacerbates viral disease severity (6, 7, 24, 25, 28–30). To explore the impact of obesity on immune responses over time, we utilized West Nile virus (WNV).

WNV is a positive-sense, single-stranded RNA member of the *Flaviviridae* family (31). WNV cycles between birds and mosquitoes with other infections, including those of humans and mice, being incidental. In humans, WNV infections are commonly asymptomatic but can cause severe illness resulting in encephalitis and meningitis (32). Protection against WNV infection is mediated by humoral and cellular immune responses (33–37). Elevated risk of severe WNV disease is associated with age, diabetes, hypertension, kidney disease and immune deficiencies. As WNV is a neurovirulent virus, severe disease in both humans and mice is associated with dissemination of the virus into the central nervous system (CNS) (33, 38–43).

Here, we show that obesity-associated chronic inflammation dysregulates host immune responses, increasing host susceptibility to severe WNV infection. We identified an early impact of obesity on viral control, where obese female mice have significantly higher viral loads in the CNS and die at a higher rate when compared to non-obese controls. Additionally, this study demonstrates that the impact of obesity on immune cell dysfunction is exacerbated in obese female mice when compared to obese male mice, as highlighted by significant defects in the ability of neutralizing antibodies primed in obese female mice to limit WNV infection. Overall, our data reveal that obesity has an impact early during the course of infection in inducing dysfunctional immunological responses to WNV infection.

## Materials and Methods

### Ethics Statement

All animal studies were conducted in accordance with the National Institutes of Health Guide for Care and Use of Laboratory Animals and approved by the Saint Louis University Animal Care and Use Committee (IACUC protocol 2771).

### Virus and Cells

WNV (strain New York 99) was passaged once in Vero cells (African green monkey kidney epithelial cells) purchased from American Type Culture Collection (ATCC CCL-81). Virus was titered *via* focus forming assay (FFA) on Vero cells as previously described (44).

### Mice and Viral Infections

Wild type C57BL/6J mice were purchased commercially from Jackson Laboratories and housed in a pathogen-free mouse facility at the Saint Louis University School of Medicine. 3- to 12-week-old mice were fed either a control (wt) or high fat diet (HFD) (40% kcal fat, 20% kcal fructose and 2% cholesterol, Research Diets Inc.) for approximately 12 weeks. Once mice on the HFD weighed 25% more than wild type counterparts, they were considered to be obese (ob). 15- to 30-week-old male and female C57BL/6J mice were infected subcutaneously (SC) *via* footpad injection with 100 FFU of WNV.

### Measurement of Liver Enzyme Levels

Serum was collected from naïve wild type and obese mice that had been fed their respective diets for 12 weeks. Serum was diluted 1:2 in PBS and loaded into a sample collection cup for analysis *via* an IDEXX Catalyst One Chemistry Analyzer. An NSAID clip was loaded into the analyzer to measure liver damage based on serum levels of alkaline phosphatase, alanine aminotransferase and aspartate aminotransferase.

### Measurement of Circulating Inflammatory Cytokine Levels

Blood from naïve wild type and obese female and male mice that had been fed their respective diets for 12 weeks was collected into RNAzol BD (Molecular Research Center, Inc.: RB 192) and RNA was isolated according to the manufacturer’s instructions. mRNA expression of TNF-α, IL-1β and IL-6 was determined through qRT-PCR using Taqman primer probe sets purchased from Integrated DNA Technologies (IDT) based on the following assay identifiers: Mm.PT.58.12575861 (TNF-α), Mm.PT.58.41616450 (IL-1β) and Mm.PT.58.10005566 (IL-6). Relative expression of each cytokine was determined by 2^ΔΔCT^ with fold induction being relative to GAPDH (assay identifier: Mm.PT.39a.1) levels of the same samples.

### Measurement of Viral Burden

15- to 17-week-old male and female C57BL/6J mice were infected SC *via* footpad injection with 100 FFU of WNV. At 3, 8 and 15 days-post-infection (DPI), intracardiac perfusion with 20 ml of PBS was performed and brains, kidneys, spleens, livers and fat were snap frozen in Sarstedt tubes. Organs were homogenized in DMEM supplemented with 5% FBS using a BeadMill 24 (Fisher Scientific). Infectious viral load was determined by incubating ten-fold serial dilutions of organ homogenate on Vero WHO cell monolayers in 96-well plates. Cells and organ homogenates were incubated for 1 hour at 37°C, then overlaid with 2% methylcellulose diluted in 5% DMEM to prevent indiscriminate viral spread. Cells were then incubated for 24 hours at 37°C prior to being fixed with 5% paraformaldehyde in PBS for 30 minutes at room temperature. Cells were then washed 3x with PBS and permeabilized for 10 minutes in focus forming assay permeability (FFA perm) wash (PBS, 0.05% Triton-X). Foci formed from infected cells were identified by incubating plates with anti-mouse 4G2 (D1-4G2-4-15) (BioXCell), a flavivirus group antibody that binds to the fusion loop of domain II on the envelope protein, at 1 µg/ml for 2 hours at room temperature. Cells were washed 3x with FFA perm wash and incubated for 1 hour at room temperature with a goat anti-mouse horseradish peroxidase-conjugated (HRP) secondary antibody (Sigma) at 5µg/ml. Cells were again washed 3x with FFA perm wash and TrueBlue detection reagent (KPL) was added for visualization of foci of infection. Foci were counted using a CTL Elispot as described in (45). For quantification of viral genome copies, RNA was extracted from organ homogenates using TriReagent RT (Molecular Research Center Inc.: RT111). Viral genome copies were quantified *via* qRT-PCR using Prime-Time primer-probe sets purchased from IDT with the following sequences: Forward: TCAGCGATCTCTCCACCAAAG, Reverse: GGGTCAGCACGTTTGTCATTG, Probe: TGCCCGACCATGGGAGAAGCTC. Viral genome copies/µl were quantified based on a standard curve generated through dilutions of a flavivirus copy control.

### Focus Reduction Neutralization Tests

Mouse serum was diluted 1:10 in PBS, serially diluted 3-fold and mixed with ~100 FFU of WNV. Serum:virus dilutions were incubated for 1 hr at 37°C to allow for immune complex formation. Complexes were then added to Vero WHO cell monolayers in 96-well plates and incubated for 1 hr at 37°C to allow for viral entry into cells. Cells were then overlaid with 2% methylcellulose, and plates were incubated 24 hr at 37°C. Plates were fixed with 5% paraformaldehyde for 30 minutes at room temperature. Plates were then rinsed with PBS and permeabilized for 10 minutes with FFA perm wash. Foci formed from infected cells were detected by incubating cells with α-mouse 4G2 (D1-4G2-4-15) at 1µg/ml for 2 hours at room temperature. Plates were washed 3 times with FFA perm wash and incubated for 1 hr at room temperature with goat α-mouse HRP-conjugated secondary (Sigma) at 5µg/ml. Plates were then washed 3 times with FFA perm wash and foci were visualized through the addition of TrueBlue detection reagent (KPL). Foci were counted using a CTL Elispot. Neutralization curves and FRNT_90_ and FRNT_50_ values were generated in GraphPad Prism 8 though x-axis logarithmic transformation followed by a non-linear curve fit regression analysis.

### Statistical Analyses

All statistical analyses were performed using GraphPad Prism 8. Survival curve statistical differences were determined using Mantel-Cox tests. Statistical differences in weight gain, liver enzyme levels, viral burden and neutralizing antibody FRNT_90_ and FRNT_50_ values were determined by Mann-Whitney tests.

## Results

### Diet-Induced Obese Mice Display Liver Damage and Elevated Inflammatory Cytokine Levels

**Figure 1:**
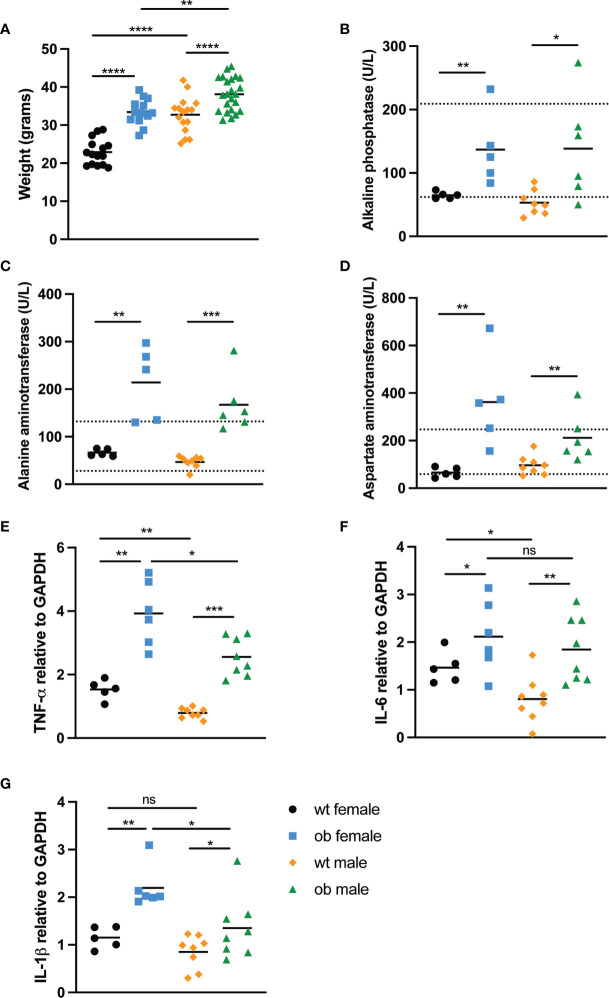
Diet-induced obese mice experience liver damage and have elevated levels of circulating inflammatory cytokines. **(A)** Weights at time of infection. To ensure mice fed the high fat diet were obese prior to infection, female (wt n=28 and ob n=27) and male (wt n=20 and ob n=27) mice were weighed. Both female and male ob mice weighed significantly more than wt counterparts (p<0.0001). **(B–D)** Serum liver enzyme levels. Liver function was monitored by measuring serum levels of **(B)** alkaline phosphatase (ALKP), **(C)** alanine aminotransferase (ALT) and **(D)** aspartate aminotransferase (AST) in wt female (n=5), ob female (n=5), wt male (n=8) and ob male (n=6) mice. All liver enzyme levels tested were detected at significantly higher amounts in ob mice when compared to wt mice, and numerous ob mice displayed enzyme levels outside of the normal range for a mouse (as indicated by the dotted lines). When comparing ALKP levels in ob versus wt females, p=0.0079, while p=0.0127 for ob versus wt males. When comparing ALT levels, p=0.0079 for ob versus wt females, while p=0.0007 for ob versus wt males. When comparing AST levels, p=0.0079 for ob versus wt females, while p=0.0080 for ob versus wt males. **(E–G)** Circulating inflammatory cytokine levels. qRT-PCR was used to characterize expression of three inflammatory cytokines, tumor necrosis factor-α (TNF-α), interleukin-6 (IL-6) and interleukin-1β (IL-1β), in the blood of naïve wt and ob female (n=5 and n=6, respectively) and male (n=8 and n=8) mice. In each instance, inflammatory cytokine levels were significantly higher in ob mice when compared to wt counterparts. Additionally, overall, inflammation was higher in female mice when compared to male mice. **(E)** TNF-α expression: p=0.0043 for ob versus wt females, p=0.0047 for ob versus wt males, p=0.0080 for ob females versus ob males and p=0.0109 for wt female versus wt males. **(F)** IL-6 expression: p=0.0303 for ob versus wt females, p=0.0045 for ob versus wt males, p=0.0533 for ob females versus ob males and p=0.0109 for wt female versus wt males. **(G)** IL-1β expression: p=0.0303 for ob versus wt females, p=0.0499 for ob versus wt males, p=0.0127 for ob females versus ob males and p=0.0109 for wt female versus wt males. Statistical significance was determined by Mann-Whitney test. *p < 0.05, **p < 0.01, ***P < 0.001, ****p < 0.0001, ns, not significant.

To determine the impact of obesity on immune responses to WNV, we developed a mouse model of high fat diet induced obesity based on previously published studies (46, 47). Female and male 3-5-week-old C57BL/6J mice were fed either a regular chow diet (wild type) or high fat diet. Mice were considered obese (ob) when they weighed 25% more than wild type (wt) mice, which occurred approximately 12 weeks after initial high fat diet feeding (p<0.0001) (**Figure 1A**). As wild type and ob female mice weighed significantly less than their male counterparts (p <0.0001 for wild type mice and p=0.0018 for obese mice), we separated the mice based on sex. The significant elevation in weight observed in ob female and ob male mice compared to the wild type controls has been shown to be a reasonable surrogate for obesity as determined by elevated BMI in humans (48, 49).

Due to diet-induced obesity being linked to nonalcoholic fatty liver disease (50, 51), we sought to determine if liver-resident enzyme levels were increased in serum of obese mice. When wild type and obese mice had been fed their respective diets for 12 weeks, serum was collected to measure circulating levels of alkaline phosphatase, alanine aminotransferase and aspartate aminotransferase (**Figures 1B–D**). Obese mice displayed significantly increased alkaline phosphatase (female p=0.0079 and male p=0.0127), alanine aminotransferase (female p=0.0079 and male p=0.0007) and aspartate aminotransferase (female p=0.0079 and male p=0.0080) levels when compared to wild type mice. The elevated liver enzyme levels in the obese mice indicate hepatocyte death due to normally liver-resident enzymes being released into the bloodstream. Importantly, obese liver enzyme levels often fell outside of the normal range for mice as indicated by the dotted lines, suggesting that the high fat diet fed mice exhibited evidence of disease similar to nonalcoholic fatty liver disease seen in humans (52, 53).

Since human obesity is accompanied by chronic inflammation, we also sought to measure circulating inflammatory cytokine levels of the mice in our model. We noted that obese female mice displayed significantly higher TNF-α (p=0.0043), IL-6 (p=0.0303) and IL-1β (p=0.0043) levels when compared to female wild type counterparts (**Figures 1E–G**). Similarly, obese males also displayed significantly higher circulating levels of TNF-α (p=0.0002), IL-6 (p=0.0019) and IL-1β (p=0.0499) when compared to wild type male counterparts (**Figures 1E–G**). Interestingly, wild type females also displayed significantly higher levels of TNF-α (p=0.0016) and IL-6 (p=0.0186) when compared to wild type males, while obese females displayed significantly higher levels of TNF-α (p=0.0200) and IL-1β (p=0.0127) when compared to obese males (**Figures 1E–G**). These data highlight that inflammation is generally higher in females than in males, regardless of diet status. Taking together the sex differences in terms of weight change and inflammation levels, we decided to separate the female and male mice for the remainder of our studies.

### Obesity Enhances the Mortality Rate of Female Mice Following WNV Infection

**Figure 2:**
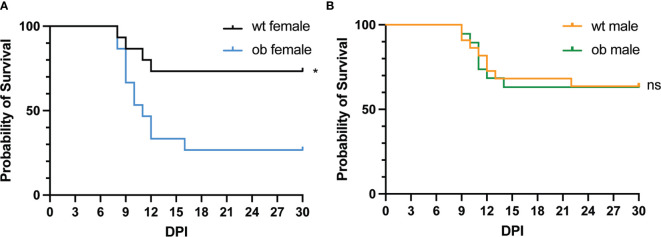
Diet-induced obese mice have enhanced mortality following viral infection. **(A, B)** Survival of mice following WNV infection. Mice were infected with 100 FFU of WNV *via* subcutaneous foot pad injection. **(A)** Wt females (n = 15) displayed a significantly higher survival rate when compared to ob females (n = 15) (p = 0.0132). **(B)** No differences were noted in the survival rates between male wt and ob mice (n = 22 and n = 19, respectively) (p = 0.9559). Survival significance was determined *via* Mantel-Cox test. *p < 0.05, ns, not significant.

Following the establishment of our obesity mouse model, we sought to test our hypothesis that obese mice would be more susceptible to severe WNV infection. To test this, female and male obese and wild type mice were infected subcutaneously (SC) with 100 focus forming units (FFU) of WNV *via* hind footpad injection. Following infection, the mice were monitored for survival for 30 days. Wild type females exhibited a significantly higher survival rate when compared to obese females with ~73% of wild type females surviving, while only about ~26% of obese females survived (p=0.0132) (**Figure 2A**). The mean time to death (MTD) for the obese females was 10 days post infection (DPI). These findings are consistent with studies done using influenza viral infection models in obese mice (25, 28, 54, 55). However, no significant differences were noted in survival rates between wild type and obese male mice (**Figure 2B**). Wild type females also displayed a 10% greater survival rate than wild type males (**Figures 2A, B**). The disparity between the survival rate of male and female mice was somewhat surprising, although studies in humans have previously noted differences in WNV disease severity between sexes (56, 57).

### Obesity Enhances WNV Load in the CNS of Female Mice

**Figure 3:**
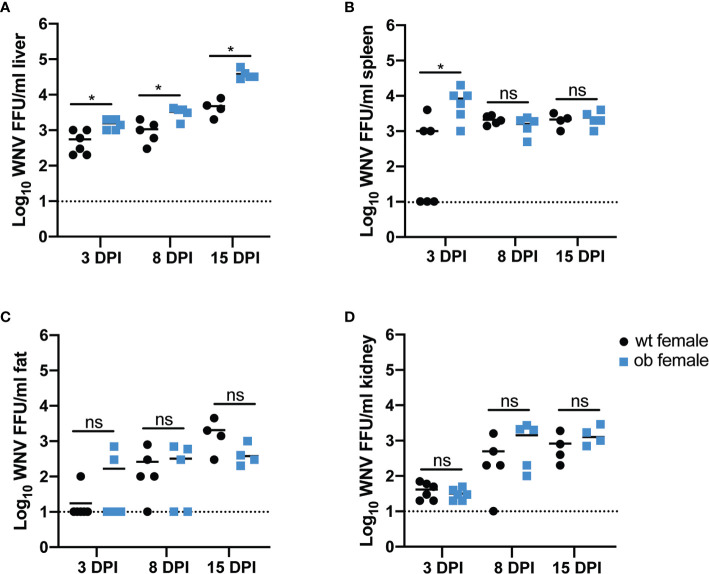
The obese state in females promotes early WNV entry into peripheral organs. **(A–D)** Infectious viral titers in female mouse organs. Mice were infected with 100 FFU of WNV *via* subcutaneous foot pad injection. At 3 (wt n=6 and ob n=6), 8 (wt n=5 and ob n=5) or 15 (wt n=4 and ob n=4) days post infection, liver **(A)**, spleen **(B)**, fat **(C)** and kidney **(D)** were harvested, frozen and homogenized. Levels of infectious virus were measured *via* focus forming assay and reported as FFU/ml of organ homogenate. Asterisks indicate statistically significant values (*p < 0.05) as determined by Mann-Whitney test. ns, not significant.

Due to the high mortality rate of obese females, we next sought to measure viral titers in various organs previously shown to harbor productive WNV replication (58) to determine if increased viral burden contributed to enhanced mortality rates or altered WNV tissue tropism. To this end, wild type and obese mice were infected SC with 100 FFU WNV. The following organs were collected at 3, 8 and 15 DPI: subcutaneous fat, liver, spleen, kidney and brain. As we noted a significant difference in the survival only with the female mice (**Figure 2**) we separated the analysis of the mice based on the sex of the animals. Virus was quantified both by focus forming assay to detect infectious virus (**Figure 3**), and qRT-PCR to detect WNV genome copies (**Supplementary Figure 1**).

In the periphery, obese females exhibited a slight but significantly higher level of infectious WNV in the liver at each time point post infection (3 DPI p=0.0433, 8 DPI p=0.0238, 15 DPI p=0.0286) (**Figure 3A**). Similarly, the female obese state displayed higher levels of infectious WNV in the spleen at 3 DPI (p=0.0152) when compared to wild type females, however, no differences in titer were noted at 8 or 15 DPI (**Figure 3B**). The infectious virus was not significantly different in other peripheral sites including fat and kidney at any time point post infection (**Figures 3C, D**). Examination of the WNV genome copies in the peripheral organs showed no differences between the obese and wild type female mice liver and fat titers (**Supplementary Figures 1A, C**), but a slight yet significantly lower amount of viral genome copies was observed in the obese mouse spleens (p=0.0315) and kidneys (p=0.0268) at eight days post infection (**Supplementary Figures 1B, D**). As expected, WNV viral titer data showed no differences in infectious virus or viral genome copies between male wild type and obese mice in any peripheral or CNS organ at any time point tested (**Supplementary Figures 2**, **3**). Overall, these data suggest that the obese state in female mice may contribute to modestly higher viral replication in some peripheral organs with the greatest impact occurring in the liver, but the slight differences in viral titer in the periphery do not appear to explain the stark differences in mortality between the obese and wild type female mice.

**Figure 4:**
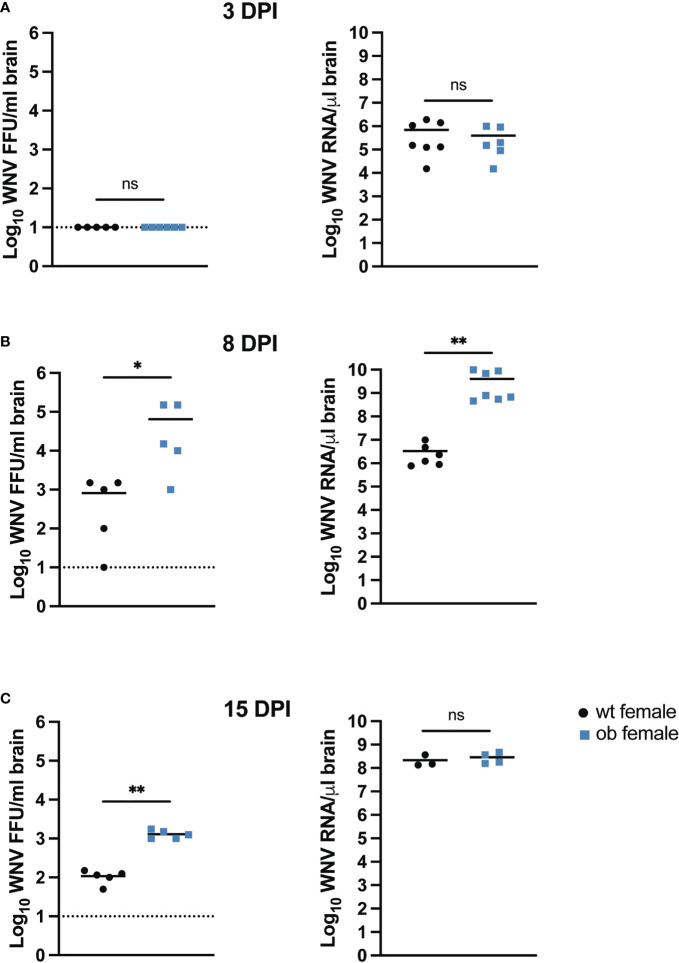
The obese state in female mice promotes heightened viral titers in the brain. **(A–C)** Brain viral titers in female mice. Mice were infected with 100 FFU WNV *via* SC footpad injection and brains were harvested at 3 **(A)**, 8 **(B)** and 15 **(C)** DPI. Brains were homogenized and used to determine infectious titer *via* focus forming assay or RNA was isolated and used for qRT-PCR analysis to determine WNV genome copy numbers based off a copy control. Infectious titer data were reported as WNV FFU/ml and genome copy data were reported as WNV RNA/µl based off GAPDH expression. Asterisks indicate statistically significant values (*p < 0.05, **p < 0.001) as determined by Mann-Whitney test. ns, not significant.

As WNV is predominately a CNS disease (59) we hypothesized that the higher mortality observed in the obese females would be associated with higher WNV titers in the brains of these animals. To determine if obesity impacted viral infection in the CNS, we examined WNV viral titer in the brains of the obese and wild type female mice at 3, 8 and 15 DPI (**Figure 4**). We were unable to detect infectious virus and saw no differences in the genome copy numbers between the wild type and obese females at 3 DPI (**Figure 4A**). However, by 8 DPI, obese females had approximately two logs more infectious virus in the brains as compared to the wild type control mice (p=0.0397) (**Figure 4B**). The obese female mice also displayed significantly higher WNV genome copies in the brain at this time point (p=0.0012) (**Figure 4B**). The obese female mice maintained a significantly higher infectious viral titer compared to the controls with a log difference between the two groups in the surviving mice at 15 DPI (p=0.0079) (**Figure 4C**), but there were no differences in the genome copy numbers of WNV in brains between these groups at this time point. Interestingly, the genome copy number in the brains of the wild type animals increased between day 8 and 15 post infection. Importantly, as the MTD for the obese mice is 10 DPI, viral titer in the surviving obese mice decreased suggesting some evidence of effective immune control in the surviving animals.

### Obese Females Generate Poorly Functioning Neutralizing Antibodies to WNV

Previous studies have shown that obesity can lead to poor antibody responses following vaccination (25–27, 60–62). Therefore, we hypothesized that the increase in mortality and high viral loads seen in the obese female mice was due in part to defects in the antibody response in the obese WNV infected animals. To test this hypothesis, we analyzed the function of neutralizing antibodies in wild type and obese mice at 8, 15 and 30 DPI through focus reduction neutralization tests (FRNTs), as we described previously (63). Employing the use of FRNTs allowed us to determine the concentration of serum, as a by proxy of neutralizing antibody titer, required to neutralize 90% (FRNT_90_) and 50% (FRNT_50_) of WNV present in the assay. Between male obese and male wild type mice, the percentage of WNV infected cells was slightly elevated at low serum dilutions at 8 and 30 DPI, but as the serum dilution increased, nearly identical infection rates were noted between the two groups (**Supplementary Figures 4A, G**). In addition, both obese and wild type males display similar FRNT_90_ and FRNT_50_ values, suggesting that neutralizing antibodies primed in male obese mice are functional (**Supplementary Figures 4B, C, E, F, H, I**).

**Figure 5:**
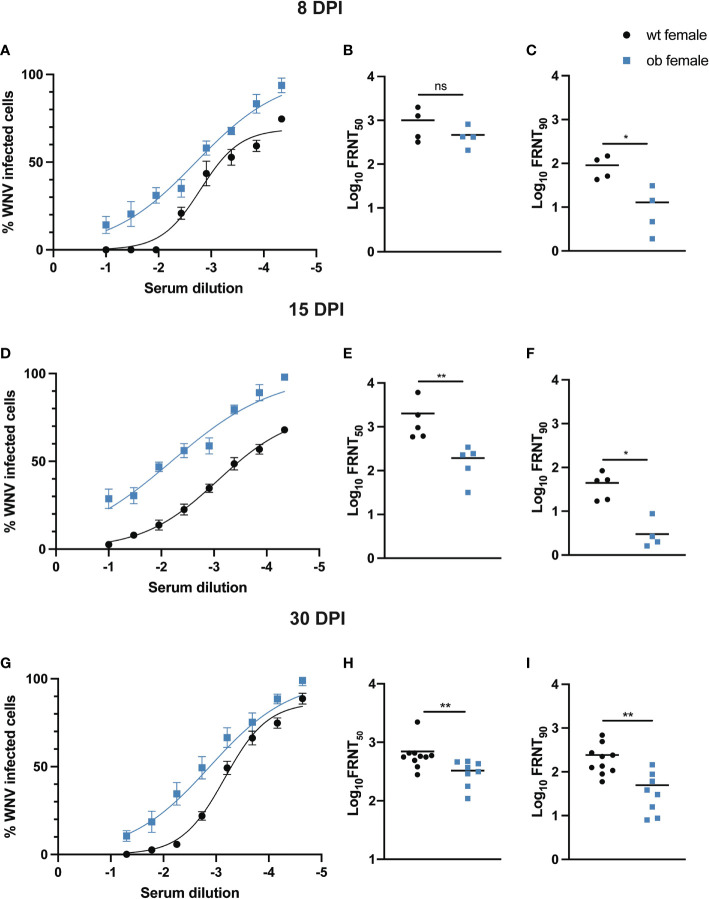
Obese females have poorly neutralizing antibodies to WNV. At 8 (wt n = 4 and ob n = 4), 15 (wt n = 5 and ob n = 5) and 30 (wt n = 10 and ob n = 8) DPI, focus reduction neutralization tests were performed to assess neutralizing antibody function. Neutralization curves at each time point **(A, D, G)** show a higher frequency of infected cells when virus was incubated with serum derived from obese females. Although no differences are noted between FRNT50 values at 8 DPI between wild type and obese animals **(B)**, these values are significantly lower in obese females at 15 and 30 DPI when compared to wild type counterparts **(E, H)**, while FRNT90 values are significantly lower in obese females when compared to wild type at each time point tested **(C, F, I)**. Asterisks indicate statistically significant values (* p<0.05, ** p<0.001) as determined by Mann-Whitney test. ns, not significant.

At 8, 15 and 30 DPI in the female mice, there is a higher percentage of WNV infected cells for each obese mouse serum dilution tested when compared to the serum dilutions of wild type females (**Figures 5A, D, G**), suggesting that the neutralizing antibodies primed in the female obese state fail to neutralize WNV as robustly as those antibodies primed in wild type females. Further, at each time point tested, FRNT_90_ values are significantly lower in the obese females when compared to the wild type females (8 DPI p=0.0286, 15 DPI p=0.0079, 30 DPI p=0.0021), again highlighting a defect in the quality of neutralizing antibodies primed in obese female mice (**Figures 5C, F, I**). Similarly, FRNT_50_ values trend toward being lower in obese females versus wild types at 8 DPI (**Figure 5B**), and these values are significantly lower in obese females at 15 and 30 DPI (15 DPI p=0.0079, 30 DPI p=0.0044) (**Figures 5E, H**). Thus, the neutralizing antibodies primed in the obese females display a reduced neutralization capacity when compared to those primed in the wild type females.

## Discussion

With obesity rates rising globally and having links to numerous pathophysiological conditions, we sought to determine if obesity conferred immunological dysfunction in a murine diet-induced obesity WNV infection model. Here we have shown that in female mice, high fat diet feeding induces an obese state that promotes increased mortality, heightened viral titers in the brain, and impaired function of neutralizing antibodies. In this report, we primarily focused on the female data as it highlights the most pronounced differences in disease, but current studies are underway in our laboratory to further investigate the interplay between chronic inflammation and sex in altering immune responses to viral pathogens. Interestingly, sex has been cited as a confounding factor in immune responses to viral pathogens with males generally having a higher risk of severe outcomes from respiratory infections at younger and older ages, while females are often at a higher risk for severe viral disease during reproductive years (reviewed in [64)].

We noted a stark difference in survival between wild type and obese females where obese females died at a significantly higher rate than wild type females (**Figure 2A**). The WNV titers in the peripheral organs revealed modest differences between the obese and wild type female mice with elevated titers in the livers and spleens of obese females at various earlier time points post infection (**Figures 3A, B**). These findings are consistent with other studies exploring the impact of obesity on organ titer in the context of various viral infections (55, 65, 66). As WNV is known to be neuroinvasive (67, 68), we sought to explore a potential role of obesity in altering WNV pathogenesis. Notably, the obese females displayed significantly higher titers in the brains at 8 and 15 DPI when compared to wild type females (**Figures 4B, C**). As shown previously by Brien et al., elevated WNV titers in the brain significantly correlate with mortality from WNV (69). Based on our work and on previous studies in the literature, we can conclude that the significant increase in mortality in the obese mice is due to the elevated viral titers observed.

Numerous studies exploring the effect of diet-induced obesity on vaccination outcomes to various viruses and toxins, including influenza and hepatitis B viruses and tetanus toxin, have highlighted that the titer of neutralizing antibodies primed in the obese state is reduced when compared to the wild type state, and such antibodies wane rapidly (26, 27, 29, 62, 70, 71). These observations highlight a potential defect in the formation of memory B cells within the obese state, a phenomenon that could be detrimental in the context of WNV infection as neutralizing antibodies are essential in reducing viral load amidst WNV infection (33, 72, 73). Previous studies have also shown that WNV specific antibody responses are important for the control of WNV burden in the CNS (33). Thus, we sought to determine if the obese state impacted the function of neutralizing antibodies. As can be seen on the neutralization curves in **Figure 5**, we noted a higher frequency of WNV-infected cells at each dilution tested from female obese mouse-derived serum at all the time points observed. Similarly, when analyzing the FRNT_90_ and FRNT_50_ values, it is evident that a significantly higher amount of female obese mouse-derived serum is required to neutralize 90%, as well as half, of the virus present when compared to serum derived from wild type female mice (**Figures 5B, C, E, F, H, I**), implicating defects in sterilizing immunity within obese females.

Through these studies, we determined that obesity induces immunological dysfunction in a murine diet-induced obesity WNV infection model. We showed that high fat diet feeding in female mice induces an obese state that promotes altered viral pathogenesis, and a decreased neutralization capacity of neutralizing antibodies. The early time points studied throughout these experiments revealed that the obese state impacts the adaptive immune responses at early time points post infection, thus shedding light on the potential for obesity to induce epigenetic changes that alter the differentiation landscape within the obese state. This phenomenon could account for why obesity tends to induce impaired memory responses. Studies to further explore the sex differences noted within our model, as well as to investigate the impact of obesity on epigenetic modification of immune cells, are undergoing.

## Data Availability Statement

The original contributions presented in the study are included in the article/**Supplementary Material**. Further inquiries can be directed to the corresponding author.

## Ethics Statement

The animal study was reviewed and approved by the Saint Louis University Animal Care and Use Committee and done in accordance with the Guide for Care and Use of Laboratory Animals.

## Author Contributions

EG and AP conceptualized the work, wrote and edited the manuscript. EG, AP, JB, ES, TS and MH aided in experimental design. TS and JB aided in FRNT data analysis. Execution of all experiments was performed by EG. AP was responsible for the support of experiments. All authors contributed to the article and approved the submitted version.

## Funding

This research was supported by the National Institutes of Health grant R0112781495 from the NIAID (NIH.gov) and a Discovery award USAMRDCPR192269 from Department of Defense.

## Conflict of Interest

The authors declare that the research was conducted in the absence of any commercial or financial relationships that could be construed as a potential conflict of interest.

## Publisher’s Note

All claims expressed in this article are solely those of the authors and do not necessarily represent those of their affiliated organizations, or those of the publisher, the editors and the reviewers. Any product that may be evaluated in this article, or claim that may be made by its manufacturer, is not guaranteed or endorsed by the publisher.

## Supplementary Material

Supplementary Figure 1 The obese state in females promotes early WNV entry into peripheral organs. **(A–D)** Organ viral titers in female mice. Mice were infected with 100 FFU of WNV *via* subcutaneous foot pad injection. At 3, 8 or 15 days post infection, liver **(A)**, spleen **(B)**, fat **(C)** and kidney **(D)** were harvested, frozen and homogenized. RNA was isolated from organ homogenates and viral genome copies were quantified *via* qRT-PCR using a standard curve to interpolate values based off a copy control. Data were then normalized to GAPDH and reported as WNV genome copies/µl of organ homogenate. Asterisks indicate statistically significant values (*p<0.05) as determined by Mann-Whitney test. ns: not significant.

Supplementary Figure 2 The obese state in males does not impact the timing or replication pattern of WNV in peripheral organs. **(A–D)** Infectious viral titers in male mouse organs. Mice were infected with 100 FFU of WNV *via* subcutaneous foot pad injection. At 3 (wt n=4, ob n=3), 8 (wt n=3, ob n=3) or 15 (wt n=3, ob n=3) days post infection, liver **(A)**, spleen **(B)**, fat **(C)** and kidney **(D)** were harvested, frozen and homogenized. Levels of infectious virus were measured *via* focus forming assay and reported as FFU/ml of organ homogenate. **(E–H)** Organ viral genome copies in male mice. RNA was isolated from organ homogenates of liver **(E)**, spleen **(F)**, fat **(G)** and kidney **(H)** and viral genome copies were quantified *via* qRT-PCR using a standard curve to interpolate values based off a copy control. Data were then normalized to GAPDH and reported as WNV genome copies/µl of organ homogenate. ns: not significant.

Supplementary Figure 3 The obese state in males does not impact the timing or replication pattern of WNV in the CNS. **(A–C)** Infectious and genome copy viral titers in male mouse brain. Mice were infected with 100 FFU of WNV *via* subcutaneous foot pad injection. At 3 **(A)**, 8 **(B)** or 15 **(C)** days post infection, brains were harvested, frozen and homogenized. Levels of infectious virus were measured *via* focus forming assay and reported as FFU/ml of brain homogenate. To determine genome copy number, RNA was isolated from brain homogenates and viral genome copies were quantified *via* qRT-PCR using a standard curve to interpolate values based off a copy control. Data were then normalized to GAPDH and reported as WNV genome copies/µl of organ homogenate. ns: not significant.

Supplementary Figure 4 Obese males have no defects in neutralizing antibody function against WNV. At 8 (wt n=3 and ob n=4), 15 (wt n=4 and ob n=3) and 30 (wt n=11 and ob n=13) DPI, focus reduction neutralization tests were performed to assess neutralizing antibody function. Neutralization curves at 8 and 30 DPI **(A, G)** show a slightly higher frequency of infected cells when virus was incubated with low serum dilutions derived from obese males, but nearly identical levels of infection are ultimately reached as the serum becomes more dilute. FRNT_50_ and FRNT_90_ values are nearly identical at each time point tested between wild type and obese male mice **(B, C, E, F, H, I)**. ns: not significant.
