# Supplementary figures and images for: Obesity Enhances Disease Severity in Female Mice Following West Nile Virus Infection
Source: Front Immunol. 2021 Aug 31;12:739025. doi: 10.3389/fimmu.2021.739025 (PMC8439568; doi:10.3389/fimmu.2021.739025)

**A**

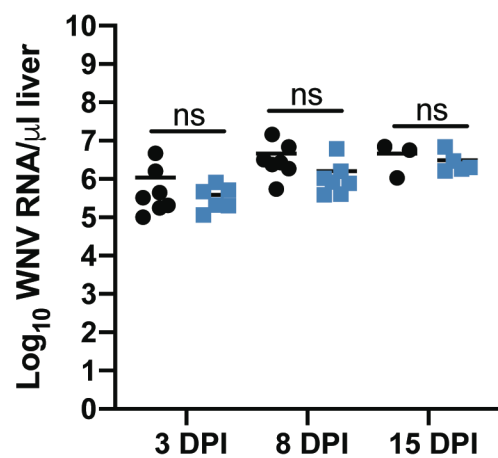

**B**

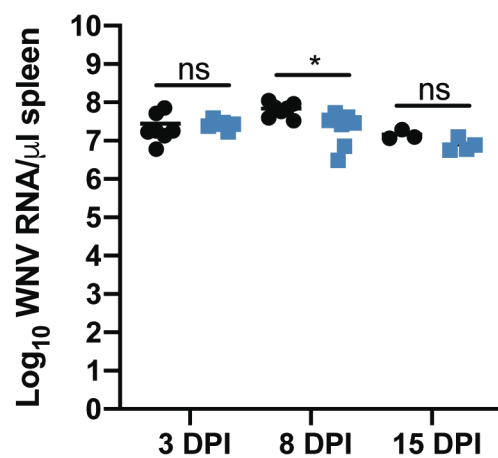

**C**

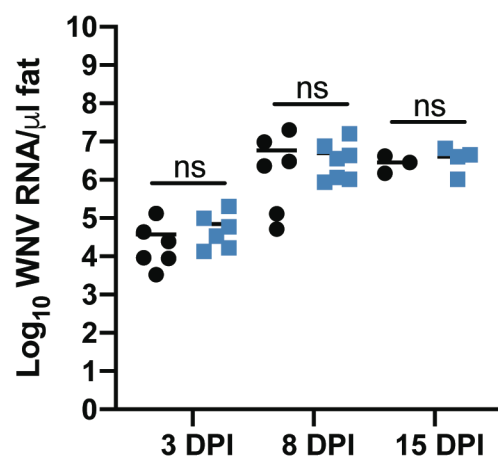

**D**

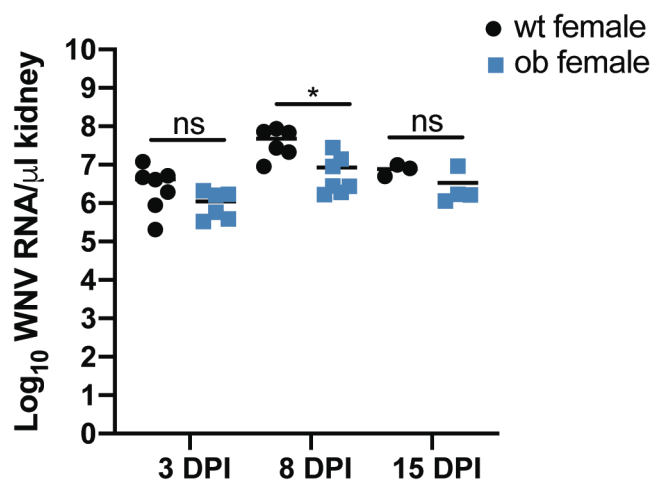

Supplement: Supplementary Figure 1 — The obese state in females promotes early WNV entry into peripheral organs. (A–D) Organ viral titers in female mice. Mice were infected with 100 FFU of WNV via subcutaneous foot pad injection. At 3, 8 or 15 days post infection, liver (A), spleen (B), fat (C) and kidney (D) were harvested, frozen and homogenized. RNA was isolated from organ homogenates and viral genome copies were quantified via qRT-PCR using a standard curve to interpolate values based off a copy control. Data were then normalized to GAPDH and reported as WNV genome copies/µl of organ homogenate. Asterisks indicate statistically significant values (*p<0.05) as determined by Mann-Whitney test. ns: not significant. [file Image_1.pdf]

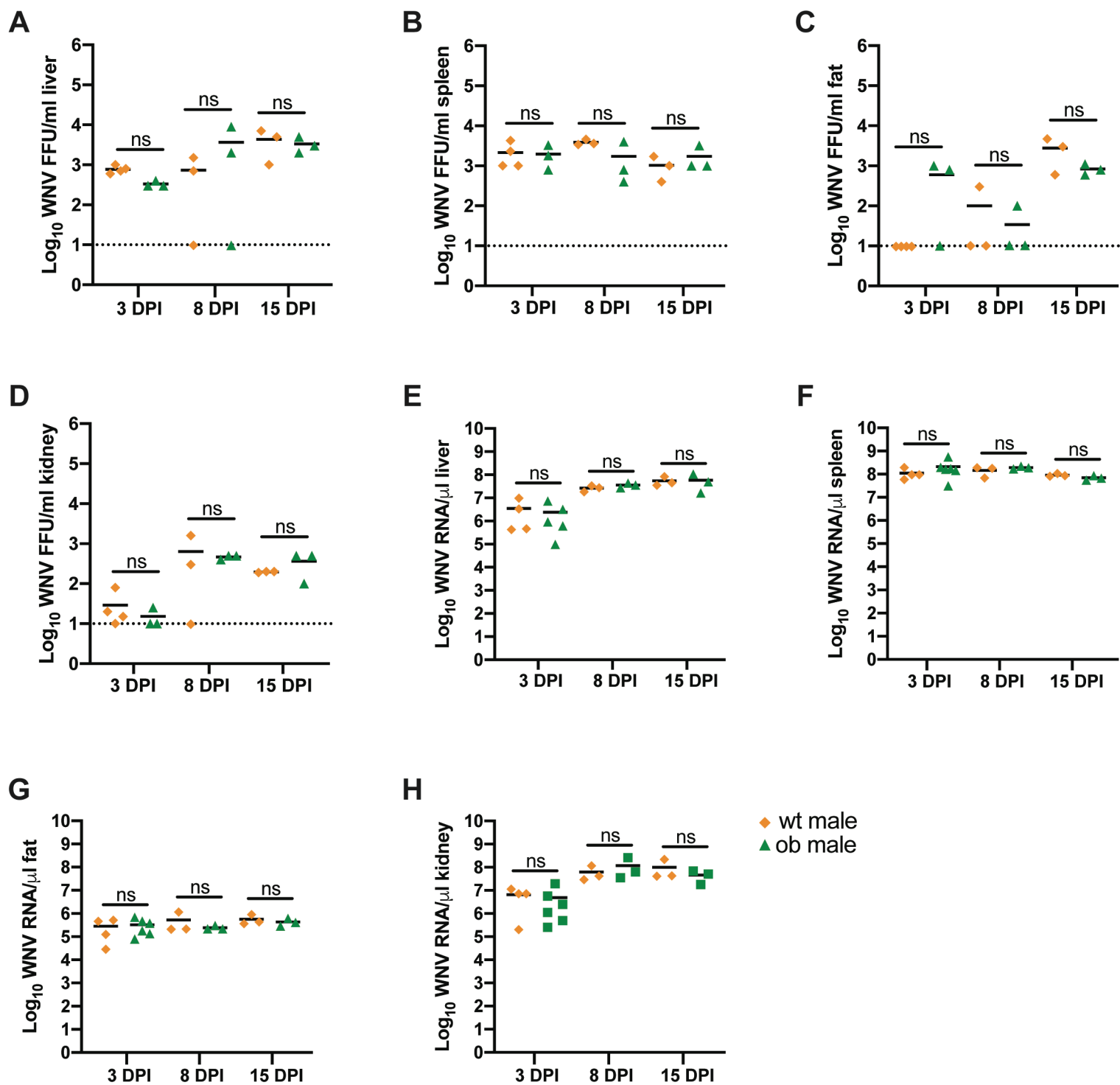

Supplement: Supplementary Figure 2 — The obese state in males does not impact the timing or replication pattern of WNV in peripheral organs. (A–D) Infectious viral titers in male mouse organs. Mice were infected with 100 FFU of WNV via subcutaneous foot pad injection. At 3 (wt n=4, ob n=3), 8 (wt n=3, ob n=3) or 15 (wt n=3, ob n=3) days post infection, liver (A), spleen (B), fat (C) and kidney (D) were harvested, frozen and homogenized. Levels of infectious virus were measured via focus forming assay and reported as FFU/ml of organ homogenate. (E–H) Organ viral genome copies in male mice. RNA was isolated from organ homogenates of liver (E), spleen (F), fat (G) and kidney (H) and viral genome copies were quantified via qRT-PCR using a standard curve to interpolate values based off a copy control. Data were then normalized to GAPDH and reported as WNV genome copies/µl of organ homogenate. ns: not significant. [file Image_2.pdf]

**A****3 DPI**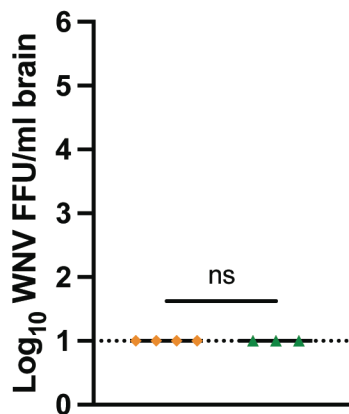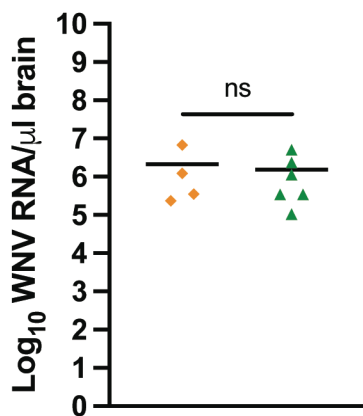**B****8 DPI**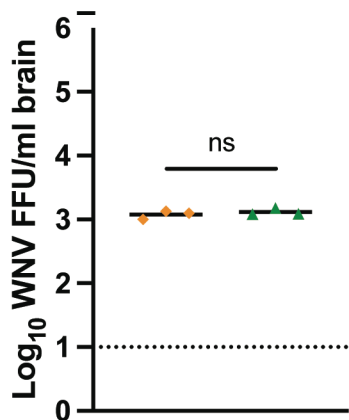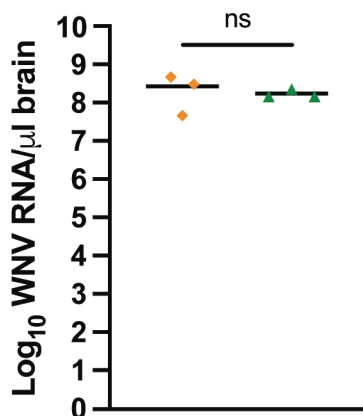**C****15 DPI**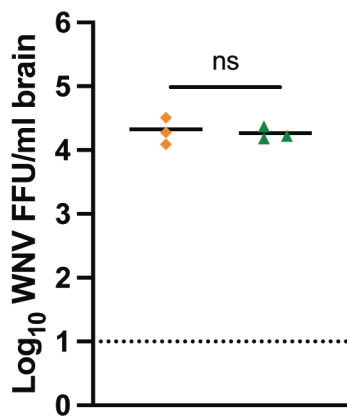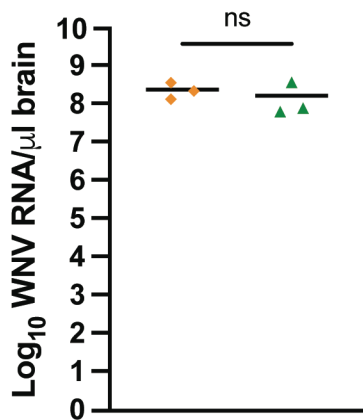

◆ wt male  
▲ ob male

Supplement: Supplementary Figure 3 — The obese state in males does not impact the timing or replication pattern of WNV in the CNS. (A–C) Infectious and genome copy viral titers in male mouse brain. Mice were infected with 100 FFU of WNV via subcutaneous foot pad injection. At 3 (A), 8 (B) or 15 (C) days post infection, brains were harvested, frozen and homogenized. Levels of infectious virus were measured via focus forming assay and reported as FFU/ml of brain homogenate. To determine genome copy number, RNA was isolated from brain homogenates and viral genome copies were quantified via qRT-PCR using a standard curve to interpolate values based off a copy control. Data were then normalized to GAPDH and reported as WNV genome copies/µl of organ homogenate. ns: not significant. [file Image_3.pdf]

8 DPI

A

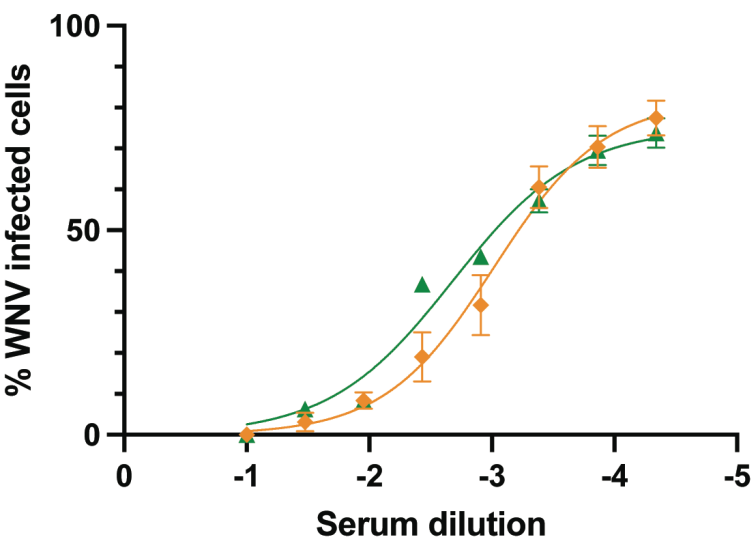

B

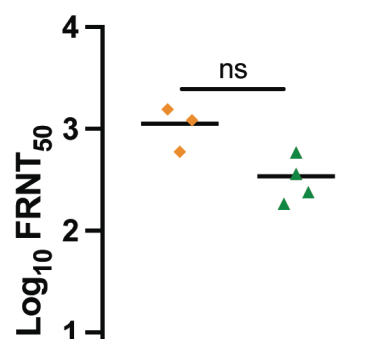

C

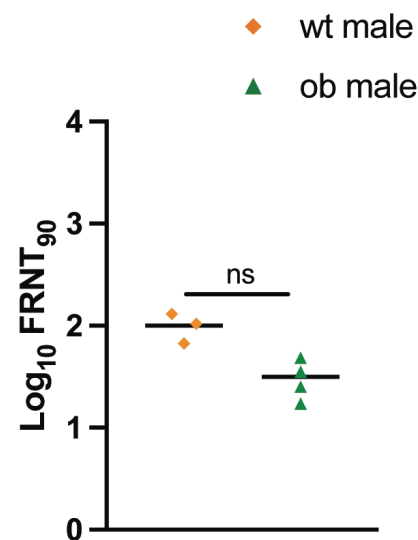

15 DPI

D

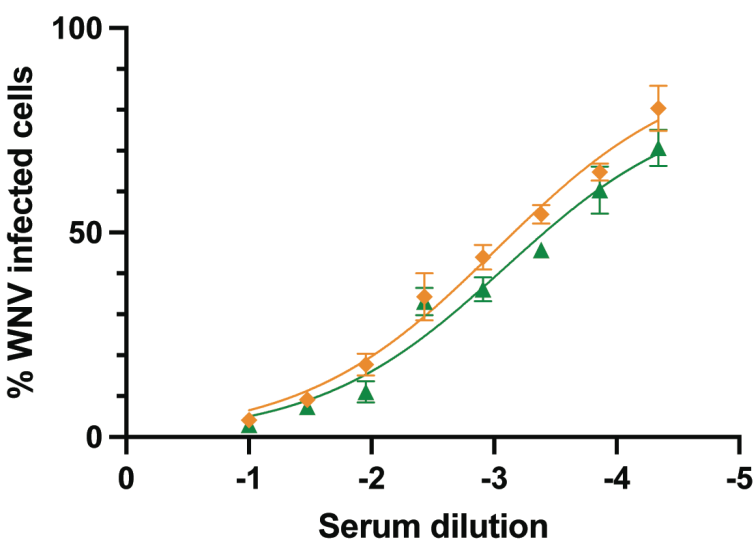

E

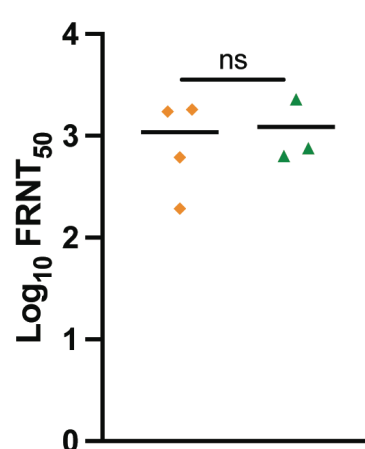

F

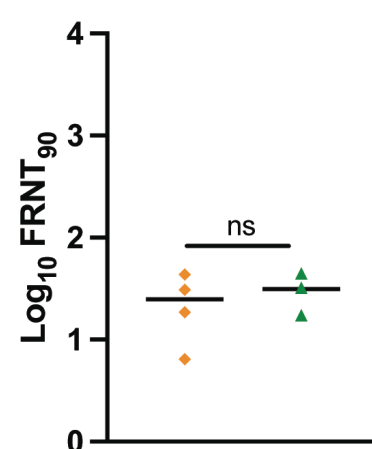

30 DPI

G

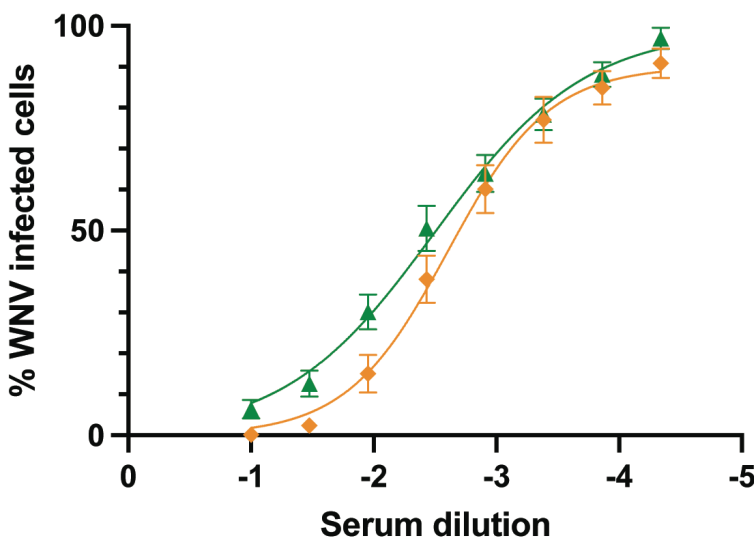

H

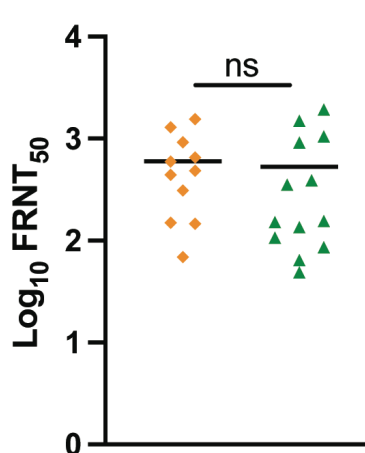

I

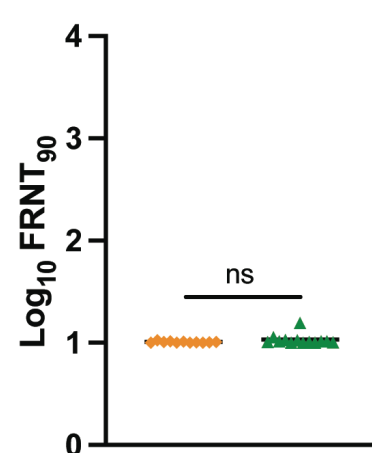

Supplement: Supplementary Figure 4 — Obese males have no defects in neutralizing antibody function against WNV. At 8 (wt n=3 and ob n=4), 15 (wt n=4 and ob n=3) and 30 (wt n=11 and ob n=13) DPI, focus reduction neutralization tests were performed to assess neutralizing antibody function. Neutralization curves at 8 and 30 DPI (A, G) show a slightly higher frequency of infected cells when virus was incubated with low serum dilutions derived from obese males, but nearly identical levels of infection are ultimately reached as the serum becomes more dilute. FRNT50 and FRNT90 values are nearly identical at each time point tested between wild type and obese male mice (B, C, E, F, H, I). ns: not significant. [file Image_4.pdf]
